# Carbon Dioxide Snow

**Published:** 1910-11-19

**Authors:** 


### SPECIAL ARTICLE.

### CARBON DIOXIDE SNOW.

Carbon dioxide snow has now been in use sufficiently long to allow of a definite opinion being expressed as to its value. There is no doubt that it constitutes a very important addition to practical therapeutics. The ease with which it can be obtained, its small cost, and the readiness with which it can be manipulated and applied are powerful arguments in its favour. Its use was preceded by that of liquid air, which, however, proved so expensive, so difficult to obtain, and so troublesome to use, that it never received extended favour, and was soon entirely superseded by the solid carbon dioxide introduced by an American surgeon for the purpose of treating moles and birthmarks.

The snow is prepared for use in the following manner: An ordinary iron cylinder of compressed carbon dioxide gas, fitted with a fine nozzle, is obtained, and over the end of the nozzle a small bag of chamois leather is fitted. It is important that the neck of this bag shall tightly embrace the metal nozzle in order to prevent the escape of gas into the external air. The stop-cock is then turned on, and in a few minutes, as the result of the extreme cold, produced by the sudden expansion of the gas, a deposit of fine floccular snow will take place inside the leather bag.

When sufficient snow has been produced, the bag is emptied and the snow placed in a brass cylinder about three inches in length, closed at one end. A small ramrod, fitting this cylinder fairly closely, is then used to press the spongy snow downwards in the cylinder. More and more snow is added until the tube is completely filled. Considerable pressure—obtained by blows of a mallet on the ramrod—will be required in order to produce solid rods of uniform diameter. The snow candle, as it is called, may be readily removed by inverting and gently tapping the cylinder, which should have its interior polished to obviate any possibility of adhesion.

The candles evaporate so slowly that they last from one to two hours, and can be used for several cases if necessary.

An ordinary penknife may be used to trim the end of the stick to any required shape or size; dependent upon the extent and nature of the area it is desired to treat.

The effect of the contact of carbon dioxide snow with the tissues is to freeze them; the depth of tissue affected depends upon the duration of contact and the degree of pressure exerted. In practice it is found that it is difficult to measure dosage by using small differences of pressure, and it is therefore measured entirely by time, a uniform pressure being exerted in all cases. This should be about 4 ounces; a little practice with the ordinary lever letter-scale will soon enable one to use this degree of pressure uniformly.

On healthy skin it is found that a short contact (fifteen seconds) produces superficial redness, followed possibly by slight blistering; a longer period (thirty seconds) produces a very superficial necrosis with a dry pale eschar; while a prolonged period (sixty seconds) produces necrosis of the whole thickness of the skin, the necrotic area again being pale, dry, and consequently aseptic throughout the process of healing.

The scarring which results is moderate in degree, and the actual scar is soft and supple in character and but little noticeable.

The pain occasioned by the application, even in such sensitive parts as the nose and face, is very slight; the average patient makes no complaint.

On removing the snow, a white, saucer-like depression, corresponding to the shape of the end of the candle, is left behind. In a few minutes, as the tissues thaw, this depression fills up again, and the skin regains its natural colour. Reaction follows after several hours, and in two or three days the amount of necrosis which is to occur will have declared itself. The parts affected should be left alone as much as possible; beyond a little powder nothing should be applied locally.

Almost all skin formations are now being treated by this method. Superficial nævi and port wine stains will be cured if the blood in their capillaries is frozen; therefore a very short application is all that is necessary. In treating large areas care should be taken that overlapping does not occur, otherwise deep scarring will result. It is best to make several separate applications every week or ten days until the whole surface has been treated.

In pigmented moles, cavernous nævi, and such conditions as lupus erythematosus, when actual destruction of the superficial tissue is necessary, an application of forty seconds is desirable. In lupus vulgaris and rodent cancer still greater depths have to be reached, and applications of from forty to sixty seconds will be necessary. It is very important in these cases to make sure that the deeper portions of the disease have been destroyed, for in both diseases the formation of healthy scar tissue is possible when there is still, in the deeper tissues, actual growth which may only manifest itself at a later date and give rise to disappointment.

It is obvious that carbon dioxide snow has a wide application. With increasing experience it is probable that it will find still wider fields of usefulness, and prove to be one of the most profitable methods we possess of dealing with superficial skin lesions which require destruction of tissue for their cure.

